# Pathogen characteristics and pre-index clinical factors associated with multidrug-resistant organism infections in intensive care unit patients

**DOI:** 10.3389/fpubh.2026.1821168

**Published:** 2026-07-30

**Authors:** Xiao-Ju Ma, Meng-Ning Chen

**Affiliations:** Department of Healthcare-Associated Infection Control, The Third Affiliated Hospital of Sun Yat-sen University, Guangzhou, Guangdong, China

**Keywords:** antimicrobial resistance, intensive care unit, logistic regression, multidrug-resistant organism, propensity score matching

## Abstract

**Background:**

Multidrug-resistant organism (MDRO) infections remain a major cause of healthcare-associated morbidity in intensive care units (ICUs). Local characterization of pathogen profiles and clinical factors associated with MDRO infection is needed to inform infection prevention and antimicrobial stewardship strategies.

**Methods:**

This single-center retrospective observational study included consecutive adult ICU admissions from January to December 2025 with an ICU length of stay ≥48 h and available microbiological cultures. MDRO infection was defined according to clinical infection criteria and phenotypic non-susceptibility to at least one agent in ≥3 antimicrobial categories. A 1:2 propensity score matching approach was used to reduce baseline imbalance. Univariable and multivariable logistic regression analyses were performed to identify pre-index clinical factors associated with MDRO infection, and model discrimination was assessed using receiver operating characteristic (ROC) analysis.

**Results:**

Among 986 eligible patients, 103 developed MDRO infection, yielding an incidence of 10.4%, with a median onset of 6 days after ICU admission. MDRO infections were mainly ventilator-associated/hospital-acquired pneumonia and bloodstream infection. Carbapenem-resistant *Acinetobacter baumannii*, carbapenem-resistant Enterobacterales, and carbapenem-resistant *Pseudomonas aeruginosa* were the predominant phenotypes. In the matched cohort (*n* = 309), pre-index clinical factors independently associated with MDRO infection included Acute Physiology and Chronic Health Evaluation II score (adjusted odds ratio [aOR] 1.04), pre-ICU antibiotic exposure (aOR 1.58), pre-index ICU carbapenem exposure (aOR 1.92), total pre-index systemic antibiotic duration in ICU (aOR 1.11 per day), pre-index mechanical ventilation (aOR 2.06), pre-index continuous renal replacement therapy (aOR 1.69), procalcitonin (aOR 1.39), and albumin (aOR 0.93). The model showed good discrimination, with an area under the ROC curve of 0.836 and a bootstrap-corrected value of 0.821.

**Conclusion:**

MDRO infections occurred in approximately one-tenth of ICU admissions and were primarily Gram-negative respiratory and bloodstream infections. Illness severity, pre-index antimicrobial exposure intensity and duration, pre-index organ support, and early inflammatory or nutritional biomarkers were independently associated with MDRO infection. These findings may support preliminary ICU risk stratification, but prospective validation and formal time-dependent exposure assessment are warranted.

## 1 Introduction

Antimicrobial resistance (AMR) has increased the burden of healthcare-associated infections (HAIs), particularly in intensive care units (ICUs), where high antimicrobial consumption, frequent invasive device use, and colonization pressure facilitate the selection and transmission of multidrug-resistant organisms (MDROs) (1, 2). Global and national surveillance reports continue to highlight HAIs and AMR as interconnected challenges requiring integrated infection prevention and control (IPC) and antimicrobial stewardship strategies (3). In ICUs, MDRO infections are especially important because delays in effective therapy and limited treatment options may contribute to adverse outcomes, while empirical broad-spectrum antimicrobial use can further promote resistance (4). Recent ICU studies have shown that carbapenem-resistant Gram-negative bacteria (CRGNB) remain prevalent and are associated with clinical and exposure-related factors, including illness severity, prior antibiotic exposure, and invasive care practices (5–7).

Respiratory and bloodstream infections are among the major clinical syndromes of MDRO infection in ICU settings. Studies of ventilator-associated pneumonia have reported substantial involvement of multidrug-resistant bacteria, supporting the role of ventilator exposure and ICU ecology in shaping MDRO risk (8). Similarly, investigations of multidrug-resistant *Pseudomonas aeruginosa* and carbapenem-resistant *P. aeruginosa* have identified invasive ventilation and antecedent antimicrobial exposure as relevant correlates of severe infection syndromes, including bloodstream infection (9). For carbapenem-resistant Enterobacterales (CRE), ongoing studies continue to define risk profiles across infection sites, reflecting the need to identify patients who may benefit from intensified surveillance and early targeted management in high-risk ICU environments (10).

Although MDRO infections in ICUs have been widely investigated, an important evidence gap remains in integrating locally relevant pathogen profiles with temporally defined assessment of clinical factors associated with clinically confirmed MDRO infection (11–13). Previous studies have often focused on overall antimicrobial resistance burden, colonization, or selected clinical variables, while less attention has been given to distinguishing infection from colonization, defining the timing of outcome ascertainment, and ensuring that ICU-acquired exposures are measured before MDRO onset (14–16). Because pathogen distribution, resistance phenotypes, antimicrobial pressure, and device-related practices vary substantially across institutions, locally derived data are needed to support targeted infection prevention and antimicrobial stewardship (17). Therefore, this study aimed to characterize the pathogen spectrum and resistance phenotypes of MDRO infections in ICU patients and to identify pre-index clinical factors associated with MDRO infection using a propensity score-matched cohort and multivariable association-based risk-stratification modeling.

## 2 Methods

### 2.1 Study design

This retrospective observational study consecutively enrolled adult patients admitted to the ICU between January 2025 and December 2025. Eligible participants were required to have (i) an ICU length of stay ≥48 h to ensure adequate exposure time for infection surveillance, and (ii) complete electronic medical records allowing extraction of demographic information, comorbidities, severity indices, antimicrobial exposure, invasive procedures, microbiological results, and clinical outcomes. Patients were included if they developed a clinically suspected or confirmed infection during ICU hospitalization with at least one corresponding microbiological culture obtained, and isolates were interpreted using routine laboratory susceptibility testing to define multidrug-resistant organism (MDRO) infection according to institutional criteria. Exclusion criteria comprised (i) age <18 years, (ii) ICU stay <48 h, (iii) readmissions during the same hospitalization (only the first ICU admission was analyzed to avoid within-patient clustering), (iv) colonization without clinical evidence of infection, defined as positive screening or culture results in the absence of compatible clinical signs, imaging findings, or treatment decision, (v) contaminated or non-diagnostic specimens (e.g., inadequate sampling or polymicrobial growth judged as contamination by the microbiology laboratory), (vi) missing key exposure or outcome variables precluding multivariable modeling, and (vii) patients transferred from other hospitals with unavailable pre-transfer antimicrobial or microbiological data when these variables were required for analysis. Informed consent was obtained from all participants and/or their legal guardians. The study was approved by the hospital ethics committee and conducted in accordance with applicable guidelines and the Declaration of Helsinki. All data were de-identified prior to analysis to protect participant confidentiality.

### 2.2 Outcome definition, grouping, and operational definitions of infection and MDRO

The primary outcome was the occurrence of MDRO infection during the ICU stay (yes/no). Patients were accordingly categorized into an MDRO infection group and a non-MDRO infection group. The outcome ascertainment date was defined as the collection date of the first clinical microbiological specimen that subsequently yielded an MDRO isolate and fulfilled the predefined clinical infection criteria. Colonization without compatible clinical evidence of infection was not considered an outcome event. Only clinical isolates obtained from specimens collected for suspected infection were included in the outcome definition; surveillance screening isolates, including rectal or perianal swabs obtained solely for colonization screening, were not used to define MDRO infection. Infection was identified based on a composite of compatible clinical features (fever and/or abnormal leukocyte count), supportive imaging and/or organ dysfunction, a clinician decision to initiate or escalate systemic antimicrobial therapy, and microbiological evidence from appropriate clinical specimens; infection syndromes were classified as ventilator-associated/hospital-acquired pneumonia (VAP/HAP), bloodstream infection (BSI), catheter-associated urinary tract infection (CAUTI), intra-abdominal/postoperative infection, and skin and soft tissue infection in accordance with CDC/NHSN criteria. MDRO was defined using standardized resistance phenotype criteria as non-susceptibility to at least one agent in ≥3 antimicrobial categories, and included clinically relevant entities such as carbapenem-resistant Enterobacterales (CRE), carbapenem-resistant *Acinetobacter baumannii* (CRAB), carbapenem-resistant *Pseudomonas aeruginosa* (CRPA), methicillin-resistant *Staphylococcus aureus* (MRSA), vancomycin-resistant *Enterococcus* (VRE), and ESBL-producing Enterobacterales.

To minimize baseline imbalance between groups, a 1:2 propensity score matching (PSM) strategy was applied, in which each patient with MDRO infection was matched to two patients without MDRO infection using nearest-neighbor matching without replacement. Propensity scores were estimated using a logistic regression model incorporating prespecified covariates available at ICU admission, including age, sex, BMI, Charlson Comorbidity Index, APACHE II score, SOFA score, primary reason for ICU admission, and major early organ dysfunction indicators (e.g., shock, acute kidney injury, and acute respiratory distress syndrome). Matching was performed using a caliper width of 0.2 standard deviations of the logit of the propensity score, and covariate balance after matching was assessed using standardized mean differences, with values <0.10 indicating acceptable balance.

### 2.3 Microbiological identification and antimicrobial susceptibility testing

Clinical specimens, including blood, sputum/endotracheal aspirates, bronchoalveolar lavage fluid (BALF), urine, drainage fluid, and wound/lesion exudates, were collected using standard aseptic techniques and processed in the hospital microbiology laboratory according to routine protocols. Bacterial species identification was performed by matrix-assisted laser desorption/ionization time-of-flight mass spectrometry (MALDI-TOF MS; Bruker Biotyper, Bruker Daltonics, Germany). Antimicrobial susceptibility testing was carried out using the VITEK 2 automated system (bioMérieux, Marcy-l’Étoile, France), and minimum inhibitory concentrations or categorical interpretations were reported by the laboratory information system. Susceptibility results were interpreted according to the Clinical and Laboratory Standards Institute (CLSI) M100 performance standards (2025 edition). Quality control was conducted throughout the study period using American Type Culture Collection (ATCC) reference strains in accordance with the manufacturer recommendations and the laboratory’s internal quality assurance procedures.

### 2.4 Data collection and definitions

Demographic variables (age, sex, and body mass index) and baseline comorbidity burden were extracted from the electronic medical record; comorbidities were summarized using the Charlson Comorbidity Index (CCI), and individual conditions of interest (e.g., diabetes mellitus, chronic kidney disease, chronic pulmonary disease, cirrhosis, malignancy, and immunosuppression) were recorded as binary variables. Illness severity and ICU admission characteristics included Acute Physiology and Chronic Health Evaluation II (APACHE II) and Sequential Organ Failure Assessment (SOFA) scores calculated within the first 24 h of ICU admission, the primary reason for ICU admission (e.g., infection, postoperative care, trauma, or neurocritical illness), and the presence of major acute complications such as shock, acute kidney injury, and acute respiratory distress syndrome. Exposure variables focused on antimicrobial and device-related factors: antimicrobial exposure before ICU admission and during ICU stay (agent classes, including carbapenems, third−/fourth-generation cephalosporins, and glycopeptides, and treatment duration in days), as well as invasive procedures and supportive therapies, including mechanical ventilation, tracheostomy, central venous catheterization, urinary catheterization, nasogastric tube placement, continuous renal replacement therapy (CRRT), extracorporeal membrane oxygenation (ECMO), and surgical drainage. For patients with MDRO infection, the index date was defined as the collection date of the first clinical specimen that subsequently yielded an MDRO isolate and fulfilled the clinical infection criteria. ICU-acquired exposures, including antimicrobial therapy, invasive devices, and organ-support interventions, were defined as exposures documented before the MDRO index date. Total systemic antibiotic duration in the ICU was calculated from ICU admission to the MDRO index date; antibiotic days after the index date were not included as antecedent exposure. Exposures initiated after the index date were not included as antecedent clinical factors in the primary association analysis. For patients without MDRO infection, exposure status and antibiotic duration were assessed within the corresponding ICU observation period using the same predefined exposure definitions. Additional treatments potentially associated with MDRO risk, including systemic corticosteroids, immunosuppressants, parenteral nutrition, and blood transfusion, were documented. Laboratory indices (white blood cell count, procalcitonin, C-reactive protein, lactate, and serum albumin) were collected at standardized time points, defined as the first available measurements within 24 h of ICU admission and, when applicable, the most recent values prior to the index infection episode. Outcome-related variables included ICU length of stay, total hospital length of stay, duration of mechanical ventilation, number of antimicrobial regimen modifications during ICU hospitalization, and treatment failure, defined as persistence or progression of infection requiring escalation of antimicrobial therapy and/or source control, development of new infection-related organ dysfunction, or infection-attributable death as adjudicated from clinical records.

### 2.5 Data management and missing data handling

All study variables were extracted from the electronic medical record, ICU information system, and microbiology laboratory database using a standardized data abstraction form. Data extraction was independently performed by two trained investigators; discrepancies were resolved by consensus after re-checking the source records, and a random sample of records was additionally audited to verify accuracy and consistency. Prior to analysis, datasets underwent range checks, logical consistency checks, and de-duplication based on unique patient identifiers. Missing data were quantified for each variable. Because several covariates had low-to-moderate missingness, multiple imputation by chained equations (MICE) was prespecified as the primary approach for the multivariable regression analysis. Twenty imputed datasets were generated under the missing-at-random assumption, and all variables used in the primary regression model, together with the outcome indicator, were included in the imputation model. Pooled estimates were obtained using Rubin’s rules. Complete-case analysis was performed as a sensitivity analysis to evaluate the robustness of the primary findings. Key variables required to define exposure status or the primary outcome (e.g., microbiological results necessary for MDRO classification, ICU admission/discharge dates, or core covariates used for model specification such as APACHE II/SOFA when mandated) were not imputed; patients with missing values for these prespecified critical fields were excluded from the corresponding analyses. Results from imputed datasets were pooled using Rubin’s rules.

### 2.6 Statistical analysis

All statistical analyses were performed using IBM SPSS Statistics, version 26.0 (International Business Machines Corporation, Armonk, NY, USA). Continuous variables were expressed as mean ± standard deviation (SD) or median [interquartile range (IQR)], and categorical variables as frequencies and percentages. Between-group comparisons were performed using Welch’s t-test, Mann–Whitney U test, Pearson’s chi-square (χ^2^) test, or Fisher’s exact test, as appropriate. A 1:2 propensity score matching (PSM) approach was used to reduce baseline imbalance, using nearest-neighbor matching without replacement and a caliper of 0.2 standard deviations of the logit of the propensity score. The multi-category variable “primary reason for ICU admission” was entered using dummy-variable coding. Covariate balance before and after matching was assessed using standardized mean differences (SMDs), with category-specific SMDs calculated for multi-category variables; an absolute SMD < 0.10 indicated acceptable balance. Univariable logistic regression was used for candidate variable screening, and variables with *p* < 0.10 or clinical relevance were entered into the multivariable logistic regression model. Adjusted odds ratios (aORs) with 95% confidence intervals (CIs) were reported. Procalcitonin was log-transformed as ln(PCT + 0.1) before modeling. Multicollinearity was assessed using variance inflation factors (VIFs), and calibration was evaluated using the Hosmer–Lemeshow test. Model discrimination was assessed using receiver operating characteristic (ROC) analysis, area under the curve (AUC), bootstrap-corrected AUC with 1,000 resamples, Brier score, and Youden index-derived diagnostic performance metrics. Multiple imputation by chained equations with 20 imputed datasets was used as the primary approach for the multivariable model, and complete-case analysis was performed as a sensitivity analysis. Pooled estimates were obtained using Rubin’s rules. Sample-size adequacy for the final model was evaluated using the events-per-variable (EPV) ratio and estimate precision. All tests were two-sided, and *p* < 0.05 was considered statistically significant.

## 3 Results

### 3.1 Study population

During January 2025 to December 2025, a total of 1,072 patients were admitted to the ICU. After screening, 986 patients met the eligibility criteria and were included in the final analytical cohort. Overall, 86 patients were excluded for the following reasons: age <18 years (*n* = 6), ICU length of stay <48 h (*n* = 28), ICU readmission during the same hospitalization (only the first ICU admission retained; *n* = 10), colonization without clinical evidence of infection (*n* = 18), contaminated or non-diagnostic specimens (*n* = 8), missing key exposure or outcome variables precluding risk-factor analysis (*n* = 12), and transfer from another hospital with unavailable pre-transfer antimicrobial or microbiological data (*n* = 4). In the final cohort (*n* = 986), 103 patients developed MDRO infection during the ICU stay and were classified as the MDRO infection group (*n* = 103). Subsequently, a 1:2 matching procedure was applied to select a comparable non-MDRO infection group (*n* = 206), yielding a matched cohort of 309 patients for between-group comparisons and downstream analyses.

### 3.2 Primary outcome incidence and infection characteristics

Among the 986 eligible ICU patients, 103 developed an MDRO infection, yielding an ICU MDRO infection incidence of 10.4% (103/986). The time from ICU admission to the first MDRO infection episode had a median of 6 [3–11] days. Within MDRO infection cases (*n* = 103), CDC/NHSN-defined infection syndromes were dominated by VAP/HAP and bloodstream infections, and the initial MDRO-defining isolates were most commonly recovered from lower respiratory tract and blood specimens. Non-fermenting Gram-negative bacilli and Enterobacterales constituted the main pathogen spectrum, with CRAB, CRE, and CRPA representing the predominant resistance phenotypes (Table 1).

**Table 1:** MDRO incidence, time to event, infection syndromes, specimen sources, pathogens, and resistance phenotypes.

Domain	Category	n/N (%) or median [IQR]
Primary outcome (overall cohort)	ICU MDRO infection incidence	103/986 (10.4)
	Time to first MDRO infection (days)	6 (3–11)
Infection syndromes (MDRO cases, *n* = 103)	VAP/HAP	45 (43.7)
	BSI	28 (27.2)
	CAUTI	14 (13.6)
	Intra-abdominal/postoperative infection	10 (9.7)
	Skin and soft tissue infection	6 (5.8)
Specimen source (index MDRO-defining specimen, *n* = 103)	Blood	28 (27.2)
	Sputum/endotracheal aspirate	30 (29.1)
	BALF	15 (14.6)
	Urine	14 (13.6)
	Drainage fluid	10 (9.7)
	Wound/lesion exudate	6 (5.8)
Infection complexity (MDRO cases, *n* = 103)	Single-site infection	85 (82.5)
	Multi-site infection	18 (17.5)
	Monomicrobial infection	79 (76.7)
	Polymicrobial infection	24 (23.3)
Pathogen spectrum (index pathogen per patient, *n* = 103)	*Acinetobacter baumannii*	32 (31.1)
	*Klebsiella pneumoniae*	24 (23.3)
	*Pseudomonas aeruginosa*	18 (17.5)
	*Escherichia coli*	10 (9.7)
	*Enterobacter cloacae complex*	5 (4.9)
	*Staphylococcus aureus*	4 (3.9)
	*Enterococcus faecium*	3 (2.9)
	*Stenotrophomonas maltophilia*	3 (2.9)
	*Proteus mirabilis*	2 (1.9)
	Other (combined)	2 (1.9)
MDRO resistance phenotypes (*n* = 103)	CRAB	32 (31.1)
	CRE	24 (23.3)
	CRPA	18 (17.5)
	ESBL-producing Enterobacterales (ESBL-E)	15 (14.6)
	MRSA	8 (7.8)
	VRE	6 (5.8)

Routine rectal surveillance swabs or active screening cultures for VRE colonization were not included in this analysis. Therefore, the VRE frequency reported in Table 1 reflects clinically identified VRE infection among MDRO infection cases rather than the overall burden of VRE colonization in the ICU. MDRO, multidrug-resistant organism; VAP/HAP, ventilator-associated/hospital-acquired pneumonia; BSI, bloodstream infection; CAUTI, catheter-associated urinary tract infection; BALF, bronchoalveolar lavage fluid; CRE, carbapenem-resistant Enterobacterales; CRAB, carbapenem-resistant *Acinetobacter baumannii*; CRPA, carbapenem-resistant *Pseudomonas aeruginosa*; ESBL-E, ESBL-producing Enterobacterales; MRSA, methicillin-resistant *Staphylococcus aureus*; VRE, vancomycin-resistant Enterococcus.

### 3.3 Baseline demographics, comorbidity burden, and ICU admission characteristics

In the 1:2 matched cohort (MDRO infection, *n* = 103; non-MDRO infection, *n* = 206), baseline demographics were comparable between groups, including age (62.5 ± 10.9 vs. 63.6 ± 11.9 years; t = −0.82, *p* = 0.416), sex distribution (male: 61.2% vs. 57.8%; χ^2^ = 0.33, *p* = 0.567), and BMI (24.4 ± 2.8 vs. 24.3 ± 3.2 kg/m^2^; t = 0.38, *p* = 0.706). Overall comorbidity burden, summarized by the Charlson Comorbidity Index, was similar (median 3 [2–4] vs. 3 [2–4]; U = 11,016, *p* = 0.577). Individual comorbidities also showed no statistically significant differences, although chronic pulmonary disease showed a borderline higher prevalence in the MDRO group (23.3% vs. 14.6%; χ^2^ = 3.64, *p* = 0.057). Regarding ICU admission characteristics, illness severity within 24 h of ICU admission was higher in the MDRO infection group, reflected by higher APACHE II scores (19.9 ± 6.5 vs. 18.0 ± 7.0; t = 2.40, *p* = 0.017) and SOFA scores (7.8 ± 3.2 vs. 6.9 ± 2.8; t = 2.32, *p* = 0.021). However, the post-matching SMDs for APACHE II and SOFA scores were both below 0.10, indicating acceptable balance according to the prespecified SMD criterion. The distribution of primary ICU admission reasons (infection, postoperative care, trauma, and neurocritical illness) was similar between groups (χ^2^ = 0.26, *p* = 0.967). Acute complications, including shock, acute kidney injury, and ARDS, were not significantly different between groups (all *p* > 0.05) (Table 2). Covariate balance before and after propensity score matching is shown in Supplementary Table S1. The multi-category variable “primary reason for ICU admission” was well balanced after matching, as reflected by similar distributions of infection, postoperative care, trauma, and neurocritical illness between groups. All covariates included in the propensity score model achieved acceptable post-matching balance, with absolute SMDs <0.10.

**Table 2:** Baseline characteristics and ICU admission features in the 1:2 matched cohort.

Variable	MDRO infection (*n* = 103)	Non-MDRO infection (*n* = 206)	Test statistic	*p* value
Age, years	62.5 ± 10.9	63.6 ± 11.9	t = −0.82	0.416
Male sex, *n* (%)	63 (61.2)	119 (57.8)	χ^2^ = 0.33	0.567
BMI, kg/m^2^	24.4 ± 2.8	24.3 ± 3.2	t = 0.38	0.706
Charlson Comorbidity Index, median [IQR]	3 [2–4]	3 [2–4]	U = 11,016	0.577
Diabetes mellitus, *n* (%)	39 (37.9)	63 (30.6)	χ^2^ = 1.65	0.199
Chronic kidney disease, *n* (%)	25 (24.3)	35 (17.0)	χ^2^ = 2.33	0.127
Chronic pulmonary disease, *n* (%)	24 (23.3)	30 (14.6)	χ^2^ = 3.64	0.057
Cirrhosis, *n* (%)	7 (6.8)	12 (5.8)	χ^2^ = 0.11	0.738
Malignancy, *n* (%)	13 (12.6)	28 (13.6)	χ^2^ = 0.06	0.813
Immunosuppression, *n* (%)	7 (6.8)	22 (10.7)	χ^2^ = 1.22	0.270
APACHE II score (within 24 h)	19.9 ± 6.5	18.0 ± 7.0	t = 2.40	0.017
SOFA score (within 24 h)	7.8 ± 3.2	6.9 ± 2.8	t = 2.32	0.021
Primary ICU admission reason, *n* (%)	Infection 46 (44.7); postoperative 30 (29.1); trauma 14 (13.6); neurocritical 13 (12.6)	Infection 93 (45.1); postoperative 63 (30.6); trauma 24 (11.7); neurocritical 26 (12.6)	χ^2^ = 0.26	0.967
Shock, *n* (%)	34 (33.0)	64 (31.1)	χ^2^ = 0.12	0.730
Acute kidney injury, *n* (%)	38 (36.9)	60 (29.1)	χ^2^ = 1.91	0.167
ARDS, *n* (%)	17 (16.5)	26 (12.6)	χ^2^ = 0.86	0.352

IQR, interquartile range; APACHE II, Acute Physiology and Chronic Health Evaluation II; SOFA, Sequential Organ Failure Assessment; ARDS, acute respiratory distress syndrome; BMI, body mass index; CCI, Charlson Comorbidity Index.

### 3.4 Pre-index exposure variables and early laboratory indices

In the 1:2 matched cohort, the MDRO group showed higher frequencies of pre-ICU antibiotic exposure and pre-index ICU broad-spectrum antibiotic exposure, particularly carbapenems and glycopeptides. Pre-index invasive device utilization and organ-support interventions were also more common in the MDRO group, including mechanical ventilation, central venous catheterization, urinary catheterization, nasogastric tube placement, CRRT, and surgical drainage. Within the first 24 h of ICU admission, the MDRO group had higher WBC counts and inflammatory biomarker levels, including procalcitonin and C-reactive protein, as well as lower serum albumin, whereas lactate levels were comparable between groups (Table 3).

**Table 3:** Pre-index exposure variables and early laboratory indices by MDRO infection status in the 1:2 matched cohort.

Variable	MDRO infection (*n* = 103)	Non-MDRO infection (*n* = 206)	Test statistic	*p* value
Pre-ICU antibiotics, *n* (%)	62 (60.2)	88 (42.7)	χ^2^ = 8.40	0.004
Pre-ICU carbapenem exposure, *n* (%)	20 (19.4)	18 (8.7)	χ^2^ = 7.26	0.007
Pre-ICU 3rd/4th-generation cephalosporin exposure, *n* (%)	25 (24.3)	32 (15.5)	χ^2^ = 3.48	0.062
Pre-ICU glycopeptide exposure, *n* (%)	12 (11.7)	10 (4.9)	χ^2^ = 4.80	0.029
Pre-index ICU carbapenem exposure, *n* (%)	71 (68.9)	92 (44.7)	χ^2^ = 16.23	<0.001
Pre-index ICU 3rd/4th-generation cephalosporin exposure, *n* (%)	55 (53.4)	78 (37.9)	χ^2^ = 6.76	0.009
Pre-index ICU glycopeptide exposure, *n* (%)	38 (36.9)	46 (22.3)	χ^2^ = 7.36	0.007
Total pre-index systemic antibiotic duration in ICU (days), median [IQR]	10.4 [6.9–14.8]	7.0 [5.0–9.7]	U = 14,576	<0.001
Pre-index mechanical ventilation, *n* (%)	82 (79.6)	118 (57.3)	χ^2^ = 15.00	<0.001
Pre-index tracheostomy, *n* (%)	18 (17.5)	20 (9.7)	χ^2^ = 3.84	0.050
Pre-index central venous catheterization, *n* (%)	90 (87.4)	160 (77.7)	χ^2^ = 4.19	0.041
Pre-index urinary catheterization, *n* (%)	95 (92.2)	172 (83.5)	χ^2^ = 4.46	0.035
Pre-index nasogastric tube placement, *n* (%)	88 (85.4)	150 (72.8)	χ^2^ = 6.18	0.013
Pre-index CRRT, *n* (%)	28 (27.2)	34 (16.5)	χ^2^ = 4.88	0.027
Pre-index ECMO, *n* (%)	6 (5.8)	4 (1.9)	Fisher’s exact	0.089
Pre-index surgical drainage, *n* (%)	22 (21.4)	24 (11.7)	χ^2^ = 5.11	0.024
Pre-index systemic corticosteroids, *n* (%)	41 (39.8)	60 (29.1)	χ^2^ = 3.56	0.059
Pre-index immunosuppressants, *n* (%)	9 (8.7)	18 (8.7)	χ^2^ = 0.00	1.000
Pre-index parenteral nutrition, *n* (%)	49 (47.6)	70 (34.0)	χ^2^ = 5.36	0.021
Pre-index blood transfusion, *n* (%)	46 (44.7)	68 (33.0)	χ^2^ = 4.00	0.045
WBC within 24 h of ICU admission (×10^9^/L), mean ± SD	13.0 ± 4.6	11.9 ± 4.0	t = 2.07	0.040
Procalcitonin within 24 h of ICU admission (ng/mL), median [IQR]	1.8 [0.7–4.1]	0.8 [0.3–2.1]	U = 13,780	<0.001
C-reactive protein within 24 h of ICU admission (mg/L), median [IQR]	100.5 [54.2–171.6]	70.4 [46.8–122.0]	U = 12,768	0.004
Lactate within 24 h of ICU admission (mmol/L), median [IQR]	2.0 [1.4–3.2]	2.0 [1.3–2.8]	U = 11,046	0.555
Albumin within 24 h of ICU admission (g/L), mean ± SD	30.7 ± 4.9	32.3 ± 4.7	t = −2.74	0.007

Unless otherwise specified, ICU-acquired exposure variables refer to exposures documented before the MDRO index date in patients with MDRO infection. Total systemic antibiotic duration in the ICU was censored at the MDRO index date, and antibiotic days after MDRO onset were not included as antecedent exposure. For patients without MDRO infection, exposure status and antibiotic duration were assessed during the corresponding ICU observation period using the same predefined exposure definitions. CRRT, continuous renal replacement therapy; ECMO, extracorporeal membrane oxygenation; IQR, interquartile range; WBC, white blood cell count.

### 3.5 Univariable analysis of factors associated with MDRO infection

Univariable logistic regression was used to identify candidate variables for multivariable modeling. Variables meeting *p* < 0.10, together with clinically relevant covariates, were carried forward to multivariable modeling. In unadjusted analyses, MDRO infection was associated with pre-ICU antibiotic exposure, pre-index ICU broad-spectrum antibiotic exposure, particularly carbapenems and glycopeptides, longer pre-index systemic antibiotic duration in the ICU, and several pre-index invasive or supportive interventions, including mechanical ventilation, central venous catheterization, urinary catheterization, nasogastric tube placement, CRRT, and surgical drainage. Higher inflammatory burden within 24 h of ICU admission, including WBC, procalcitonin, and CRP, and lower albumin was also associated with higher odds of MDRO infection, whereas lactate was not (Table 4).

**Table 4:** Univariable logistic regression for MDRO infection.

Variable	cOR	95% CI	*p* value
Pre-index antimicrobial exposure			
Pre-ICU antibiotics (yes vs. no)	2.03	1.25–3.28	0.004
Pre-ICU carbapenem exposure (yes vs. no)	2.52	1.27–5.00	0.008
Pre-ICU 3rd/4th-generation cephalosporin exposure (yes vs. no)	1.74	0.97–3.14	0.064
Pre-ICU glycopeptide exposure (yes vs. no)	2.58	1.08–6.20	0.033
Pre-index ICU carbapenem exposure (yes vs. no)	2.75	1.67–4.53	<0.001
Pre-index ICU 3rd/4th-generation cephalosporin exposure (yes vs. no)	1.88	1.17–3.03	0.010
Pre-index ICU glycopeptide exposure (yes vs. no)	2.03	1.21–3.41	0.007
Total pre-index systemic antibiotic duration in ICU (per 1 day)	1.18	1.11–1.24	<0.001
Pre-index invasive procedures/organ-support interventions			
Pre-index mechanical ventilation (yes vs. no)	2.91	1.67–5.06	<0.001
Pre-index tracheostomy (yes vs. no)	1.97	0.99–3.91	0.053
Pre-index central venous catheterization (yes vs. no)	1.99	1.02–3.88	0.043
Pre-index urinary catheterization (yes vs. no)	2.35	1.04–5.28	0.039
Pre-index nasogastric tube placement (yes vs. no)	2.19	1.17–4.10	0.014
Pre-index CRRT (yes vs. no)	1.89	1.07–3.34	0.029
Pre-index ECMO (yes vs. no)	3.12	0.86–11.33	0.083
Pre-index surgical drainage (yes vs. no)	2.06	1.09–3.89	0.026
Other pre-index treatments			
Pre-index systemic corticosteroids (yes vs. no)	1.61	0.98–2.64	0.060
Pre-index parenteral nutrition (yes vs. no)	1.76	1.09–2.86	0.021
Pre-index blood transfusion (yes vs. no)	1.64	1.01–2.66	0.046
Severity / comorbidity variables			
Chronic pulmonary disease (yes vs. no)	1.78	0.98–3.24	0.059
APACHE II score (per 1 point)	1.04	1.01–1.08	0.016
SOFA score (per 1 point)	1.10	1.02–1.18	0.017
Laboratory indices within 24 h of ICU admission			
WBC (per 1 × 10^9^/L)	1.06	1.00–1.13	0.046
Procalcitonin (per 1-unit increase in ln[PCT + 0.1])	1.62	1.29–2.04	<0.001
C-reactive protein (per 10 mg/L)	1.05	1.02–1.08	0.003
Lactate (per 1 mmol/L)	1.06	0.95–1.19	0.301
Albumin (per 1 g/L)	0.93	0.89–0.98	0.005

Unless otherwise specified, ICU-acquired exposure variables refer to exposures documented before the MDRO index date in patients with MDRO infection. Total systemic antibiotic duration in the ICU was censored at the MDRO index date, and antibiotic days after MDRO onset were not included as antecedent exposure. For patients without MDRO infection, exposure status and antibiotic duration were assessed during the corresponding ICU observation period using the same predefined exposure definitions. cOR, crude odds ratio; CI, confidence interval; CRRT, continuous renal replacement therapy; ECMO, extracorporeal membrane oxygenation; WBC, white blood cell count; PCT, procalcitonin.

### 3.6 Multivariable logistic regression for pre-index clinical factors associated with MDRO infection

In the multivariable logistic regression model, covariates were entered based on univariable analysis (*p* < 0.10) and clinical relevance. After adjustment, higher illness severity was independently associated with MDRO infection, with each 1-point increase in APACHE II score associated with 4% higher odds of MDRO infection (aOR 1.04, 95% CI 1.01–1.07, *p* = 0.010). Pre-ICU antibiotic exposure also remained independently associated with MDRO infection (aOR 1.58, 95% CI 1.02–2.46, *p* = 0.041). Pre-index ICU antimicrobial exposure was independently associated with MDRO infection, including pre-index ICU carbapenem exposure (aOR 1.92, 95% CI 1.17–3.16, *p* = 0.009) and longer pre-index systemic antibiotic duration in the ICU (per day: aOR 1.11, 95% CI 1.06–1.17, *p* < 0.001). Among pre-index organ-support variables, pre-index mechanical ventilation (aOR 2.06, 95% CI 1.20–3.55, *p* = 0.009) and pre-index CRRT (aOR 1.69, 95% CI 1.01–2.85, *p* = 0.045) were retained in the final model. Among early laboratory indicators, higher procalcitonin was associated with higher odds of MDRO infection (aOR 1.39, 95% CI 1.12–1.72, *p* = 0.002), whereas higher serum albumin was associated with lower odds of MDRO infection (per 1 g/L: aOR 0.93, 95% CI 0.89–0.98, *p* = 0.007). Multicollinearity was not evident (VIF range 1.08–2.31), and model calibration was acceptable (Hosmer-Lemeshow χ^2^ = 5.12, *p* = 0.744) (Table 5).

**Table 5:** Multivariable logistic regression for clinical factors associated with MDRO infection.

Variable	Adjusted OR (aOR)	95% CI	Wald χ^2^	*p* value
APACHE II score, per 1-point increase	1.04	1.01–1.07	6.58	0.010
Pre-ICU antibiotics, yes vs. no	1.58	1.02–2.46	4.18	0.041
Pre-index ICU carbapenem exposure, yes vs. no	1.92	1.17–3.16	6.73	0.009
Total pre-index systemic antibiotic duration in ICU, per 1 day	1.11	1.06–1.17	18.52	<0.001
Pre-index mechanical ventilation, yes vs. no	2.06	1.20–3.55	6.89	0.009
Pre-index CRRT, yes vs. no	1.69	1.01–2.85	4.01	0.045
Procalcitonin, ln[PCT + 0.1], per 1-unit increase	1.39	1.12–1.72	9.76	0.002
Albumin, per 1 g/L increase	0.93	0.89–0.98	7.28	0.007

Unless otherwise specified, ICU-acquired exposure variables refer to exposures documented before the MDRO index date in patients with MDRO infection. Total systemic antibiotic duration in the ICU was censored at the MDRO index date, and antibiotic days after MDRO onset were not included as antecedent exposure. For patients without MDRO infection, exposure status and antibiotic duration were assessed during the corresponding ICU observation period using the same predefined exposure definitions. Model diagnostics: VIF range 1.08–2.31; Hosmer–Lemeshow test χ^2^ = 5.12, *p* = 0.744. aOR, adjusted odds ratio; CI, confidence interval; APACHE II, Acute Physiology and Chronic Health Evaluation II; CRRT, continuous renal replacement therapy; PCT, procalcitonin.

### 3.7 Discriminatory performance of the exploratory risk-stratification model

**Figure 1:**
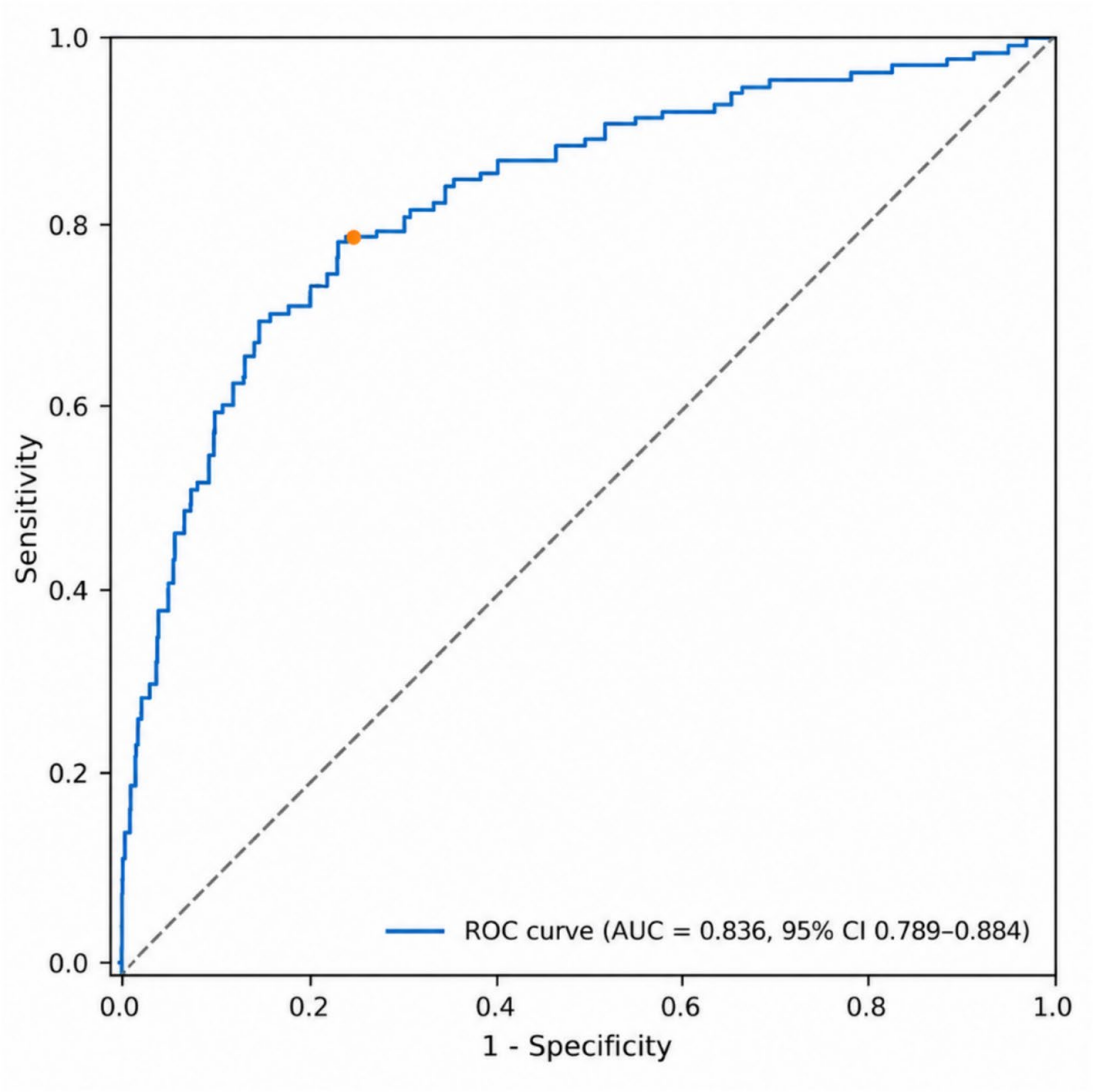
Receiver operating characteristic (ROC) curve of the final multivariable logistic regression model for predicting multidrug-resistant organism (MDRO) infection. The ROC curve was generated using predicted probabilities from the final multivariable logistic regression model in the matched cohort (*n* = 309; MDRO, *n* = 103; non-MDRO, *n* = 206). The model included APACHE II score, pre-ICU antibiotic exposure, ICU carbapenem exposure, total systemic antibiotic duration in the ICU, mechanical ventilation, continuous renal replacement therapy (CRRT), ln(PCT + 0.1), and serum albumin. The model showed good discriminatory performance, with an area under the curve (AUC) of 0.836 (95% CI, 0.789–0.884; *p* < 0.001). The orange marker indicates the optimal cut-off value (0.347) determined by the Youden index, corresponding to a sensitivity of 77.7% and a specificity of 75.2%.

ROC analysis was performed to assess the discriminatory performance of the final multivariable logistic regression model for MDRO risk stratification in the matched cohort. The model showed good discrimination, with an apparent AUC of 0.836 (95% CI, 0.789–0.884; *p* < 0.001). After 1,000 bootstrap resamples, the optimism-corrected AUC was 0.821, indicating limited optimism in internal discrimination. Using the optimal predicted-probability threshold of 0.347 determined by the Youden index, the sensitivity, specificity, positive predictive value, negative predictive value, and overall accuracy were 77.7, 75.2, 61.1, 87.1, and 76.1%, respectively. The Youden index was 0.529, and the Brier score was 0.154, suggesting acceptable overall model error. These findings indicate that the final multivariable model had acceptable-to-good ability to distinguish ICU patients with MDRO infection from those without MDRO infection in this matched cohort (Table 6; Figure 1).

**Table 6:** Discriminatory performance of the final multivariable model for MDRO risk stratification.

Performance metric	Value
Study population	Matched cohort
Sample size	309
MDRO/non-MDRO cases	103/206
Variables included in the model	APACHE II, pre-ICU antibiotics, pre-index ICU carbapenem exposure, total pre-index systemic antibiotic duration in ICU, pre-index mechanical ventilation, pre-index CRRT, ln(PCT + 0.1), albumin
Apparent AUC	0.836
95% CI for AUC	0.789–0.884
*p* value for AUC vs. 0.5	<0.001
Bootstrap-corrected AUC	0.821
Optimal cut-off value	0.347
Sensitivity	77.70%
Specificity	75.20%
Positive predictive value	61.10%
Negative predictive value	87.10%
Overall accuracy	76.10%
Youden index	0.529
Brier score	0.154

AUC, area under the receiver operating characteristic curve; CI, confidence interval; MDRO, multidrug-resistant organism; APACHE II, Acute Physiology and Chronic Health Evaluation II; ICU, intensive care unit; CRRT, continuous renal replacement therapy; PCT, procalcitonin.

Model stability and sample-size adequacy were further evaluated. The final multivariable model included eight variables and 103 MDRO infection events, corresponding to 12.9 events per variable. This events-per-variable ratio was considered acceptable for model stability. The apparent AUC was 0.836, and the bootstrap-corrected AUC after 1,000 resamples was 0.821, indicating limited optimism and stable internal discriminatory performance. Although most estimates had clinically interpretable 95% CIs, the associations for pre-ICU antibiotic exposure and pre-index CRRT should be interpreted cautiously because their lower confidence limits were close to the null value.

### 3.8 Missing data and sensitivity analyses

Overall missingness across model covariates was low to moderate and showed no clear evidence of systematic differential missingness by MDRO status. In the matched cohort (*n* = 309), the proportion of missing data for individual covariates ranged from 0.0 to 7.8%. Key outcome-defining microbiological fields required for MDRO classification were complete by design. Missingness was minimal for age and sex (0%) and low for BMI (2.6%). Severity scores had slightly higher missingness, including APACHE II (4.5%) and SOFA (4.2%). Laboratory indices within 24 h of ICU admission had the highest missingness, particularly procalcitonin (7.8%) and albumin (6.1%), whereas WBC (1.9%) and lactate (2.9%) were infrequently missing. Missingness patterns were comparable between the MDRO and non-MDRO groups, with all absolute differences <3 percentage points, which was consistent with non-systematic missingness (Supplementary Table S2).

Multiple imputation by chained equations was prespecified as the primary approach for the multivariable model presented in Table 5. Twenty imputed datasets were generated, and pooled estimates were obtained using Rubin’s rules. To assess robustness, complete-case analysis was performed as a sensitivity analysis and included 285 patients (MDRO *n* = 95, non-MDRO *n* = 190), excluding 24 patients with missing values in one or more model covariates. The direction and statistical significance of the main model variables were consistent between the imputed and complete-case models. In the complete-case analysis, APACHE II, pre-ICU antibiotics, pre-index ICU carbapenem exposure, total pre-index systemic antibiotic duration in ICU, pre-index mechanical ventilation, pre-index CRRT, ln(PCT + 0.1), and albumin showed effect estimates similar to those of the primary imputed model. No variable that was statistically significant in the primary model became non-significant in the complete-case sensitivity analysis, supporting the robustness of the findings (Supplementary Table S3).

## 4 Discussion

In this retrospective propensity score-matched study of adult ICU patients, MDRO infection occurred in 10.4% of eligible admissions, with a median onset of 6 days after ICU admission. The pathogen spectrum was predominantly Gram-negative, and MDRO infections mainly comprised VAP/HAP and bloodstream infections, with CRAB, CRE, and CRPA as the leading resistance phenotypes. After adjustment for baseline imbalance and clinically relevant covariates, pre-index clinical factors independently associated with MDRO infection included higher APACHE II score, pre-ICU antibiotic exposure, pre-index ICU carbapenem exposure, longer pre-index systemic antibiotic duration in the ICU, pre-index mechanical ventilation, pre-index CRRT, elevated procalcitonin, and lower serum albumin. The final multivariable model showed acceptable-to-good discrimination, with an apparent AUC of 0.836 and a bootstrap-corrected AUC of 0.821. The main contribution of this study lies in integrating local pathogen characteristics, clinically confirmed infection rather than colonization, temporally defined pre-index exposures, and discriminatory performance assessment within a matched ICU cohort (18, 19). Clinically, these findings may support locally informed surveillance, antimicrobial stewardship, and preliminary risk stratification of ICU patients at increased risk of MDRO infection (12, 20).

The pathogen distribution observed in this cohort provides clinically relevant information for local ICU infection prevention and empirical antimicrobial planning. MDRO infections mainly involved carbapenem-resistant Gram-negative organisms, especially CRAB, CRE, and CRPA, and were most frequently identified from respiratory and blood specimens. This distribution is consistent with the clinical profile of ICU patients, who often require airway management, mechanical ventilation, vascular access, renal replacement therapy, and repeated exposure to broad-spectrum antimicrobials. The predominance of VAP/HAP may reflect the combined background of airway instrumentation, impaired host defense, aspiration risk, prolonged critical illness, and increased airway microbial burden (21). Similarly, bloodstream infections may be related to vascular access, invasive monitoring, organ-support intensity, and systemic illness burden (22). Importantly, colonization without compatible clinical evidence of infection was not considered an outcome event, allowing the analysis to focus on clinically relevant MDRO infection rather than microbiological carriage alone (23). This distinction is important because the epidemiology and clinical implications of colonization may differ from those of confirmed infection. Therefore, the local predominance of carbapenem-resistant Gram-negative pathogens underscores the value of institution-specific microbiological surveillance and infection-control strategies tailored to the ICU pathogen ecology (24, 25).

The pre-index clinical factors retained in the final model suggest that MDRO infection in ICU patients is associated with several clinically recognizable domains, including illness severity, antimicrobial exposure burden, organ-support intensity, and early inflammatory or nutritional status. A higher APACHE II score may represent greater physiological instability and a more complex ICU course. Pre-ICU antibiotic exposure, pre-index ICU carbapenem exposure, and longer pre-index systemic antibiotic duration in the ICU may indicate greater cumulative antimicrobial exposure and more complicated preceding infectious conditions. Pre-index mechanical ventilation and pre-index CRRT may reflect both the need for advanced organ support and exposure to invasive ICU care processes. Among laboratory indicators, elevated procalcitonin may represent early systemic inflammatory burden, whereas lower serum albumin may indicate malnutrition, inflammation, capillary leakage, or reduced physiological reserve (26, 27). These variables are routinely available in ICU practice and may help clinicians identify patients who warrant closer microbiological surveillance, stricter infection-prevention measures, and more cautious antimicrobial review (28). However, given the retrospective observational design and the time-dependent nature of ICU-acquired exposures, these variables should be interpreted as clinical risk markers rather than causal determinants. Taken together, the combination of severity indices, pre-index antimicrobial exposure, pre-index organ-support variables, and early biomarkers may provide an adjunctive framework for preliminary risk stratification while awaiting microbiological confirmation (29).

Several previous studies provide useful context for interpreting the present model. Han et al. (30) analyzed pathogen distribution and factors associated with multidrug-resistant bacterial infection in ICU patients and identified mechanical ventilation and urinary catheterization as clinically relevant variables. Jiang et al. (31) developed a logistic regression-based risk-stratification model in Neuro-ICU patients and reported good discrimination, with an AUC of 0.887. Kuloglu et al. (3) focused on carbapenem-resistant Gram-negative bacterial infections and reported that colonization, previous antibiotic use, intubation, tracheostomy, and central venous catheterization were associated with infection occurrence. Our findings are directionally consistent with these studies, particularly regarding antimicrobial exposure, airway-related interventions, and invasive organ-support variables. However, our study extends prior evidence by evaluating clinically confirmed MDRO infection in a broader adult ICU cohort, excluding colonization from the outcome definition, defining ICU-acquired exposures before the MDRO index date, incorporating local resistance phenotypes, and integrating inflammatory and nutritional biomarkers. More recently, machine learning approaches have been used for ICU MDRO risk stratification. Cui et al. (32) developed models using the Medical Information Mart for Intensive Care IV (MIMIC-IV) database in ICU patients with ventilator-associated pneumonia and reported that XGBoost achieved the best test-set discrimination, with an AUC of 0.831. Zhao et al. (33) established an interpretable machine learning framework for ICU MDRO risk stratification, in which Random Forest achieved an AUC of 0.83 and SHapley Additive exPlanations (SHAP) analysis identified urinary catheterization, ventilator use, and prolonged antibiotic exposure as influential variables. In comparison, our conventional multivariable logistic regression model achieved similar discrimination, with an apparent AUC of 0.836 and a bootstrap-corrected AUC of 0.821. However, these cross-study comparisons should be interpreted cautiously because of differences in study populations, outcome definitions, predictor sets, and validation strategies. Although machine learning models may better capture nonlinear associations and complex interactions, logistic regression remains valuable in clinical settings because it provides transparent effect estimates, requires fewer routinely available variables, and is easier to reproduce at the bedside. Therefore, the present model should not be viewed as replacing machine learning approaches, but as a pragmatic and interpretable exploratory risk-stratification framework integrating local pathogen epidemiology, pre-index exposure assessment, and clinically accessible biomarkers.

The present study has practical implications for ICU infection prevention and risk assessment. Because the final model used routinely available clinical variables, it may support preliminary risk stratification without additional specialized testing. Patients with a higher model-estimated probability of MDRO infection could be prioritized for closer microbiological surveillance, antimicrobial review, reinforced contact precautions, and stricter device-management practices (34, 35). Rather than replacing microbiological diagnosis, the model may serve as an early adjunctive tool to guide intensified monitoring while awaiting definitive culture results. Methodologically, the study focused on confirmed MDRO infection rather than colonization alone, defined ICU-acquired exposures relative to the MDRO index date, and used propensity score matching to reduce baseline imbalance. Multivariable modeling incorporated severity, pre-index antimicrobial exposure, pre-index device-related and organ-support variables, and laboratory domains, while ROC analysis, bootstrap-corrected AUC, and Brier score provided discriminatory performance assessment beyond association analysis. These features support the potential utility of the model as a pragmatic ICU risk-assessment framework. However, given the retrospective observational design and the time-dependent nature of ICU-acquired exposures, the model should be interpreted as an exploratory risk-stratification tool rather than a causal model or a definitive clinical prediction instrument. Prospective external validation and formal time-dependent exposure assessment are required before broader clinical application.

Several limitations should be acknowledged. First, the retrospective design limits causal inference. Although propensity score matching, multivariable adjustment, and diagnostic checks were used to improve internal validity, propensity score matching only reduces baseline imbalance based on variables available at ICU admission and does not resolve the time-dependent nature of ICU-acquired exposures. Second, although ICU-acquired exposures were defined before the MDRO index date, variables such as antimicrobial therapy, invasive procedures, mechanical ventilation, and CRRT remain time-dependent in nature. The original retrospective dataset was not structured as a person-day longitudinal dataset, and complete day-by-day exposure status and start-stop intervals were unavailable for all relevant time-dependent variables. Therefore, this was primarily a data-structure limitation rather than only a practical analytic decision. Because logistic regression cannot fully account for changes in exposure timing, duration, or intensity, residual time-dependent bias cannot be excluded, and these variables should be interpreted as clinical risk markers rather than causal determinants. Future prospective studies with daily exposure tracking, landmark analyses, time-dependent Cox models, or marginal structural models are warranted. Third, systematic data on MDRO colonization were unavailable. Although colonization without compatible clinical evidence of infection was not considered an outcome event, we did not routinely capture MDRO colonization status at ICU admission, serial colonization during ICU stay, or unit-level colonization pressure. Therefore, we could not evaluate the transition from colonization to clinically confirmed infection or adjust for colonization as an upstream clinical factor, which is particularly relevant for carbapenem-resistant Gram-negative organisms such as CRAB and CRE. In addition, routine VRE screening or rectal surveillance cultures were not included in this retrospective analysis; therefore, the prevalence of VRE colonization and the overall VRE burden in the ICU may have been underestimated. Future studies should incorporate active surveillance cultures, including rectal or perianal surveillance for VRE when clinically indicated, and colonization-pressure measures. Fourth, the single-center design may limit generalizability to ICUs with different resistance ecology, patient populations, and antimicrobial stewardship practices. Finally, despite matching and adjustment, residual confounding from unmeasured clinical or institutional factors cannot be excluded.

## 5 Conclusion

MDRO infections occurred in approximately one-tenth of ICU admissions, typically after several days of ICU stay, and were predominantly respiratory and bloodstream infections involving Gram-negative organisms, especially CRAB, CRE, and CRPA. In the matched cohort, pre-index clinical factors independently associated with MDRO infection included higher illness severity, pre-ICU antibiotic exposure, pre-index ICU carbapenem exposure, longer pre-index systemic antibiotic duration in the ICU, mechanical ventilation, CRRT, elevated early procalcitonin, and lower serum albumin. These findings may inform preliminary risk stratification and targeted surveillance in ICU patients, while also highlighting the importance of antimicrobial stewardship, device-related infection prevention bundles, and infection-control measures directed at high-risk Gram-negative MDROs. Prospective studies with external validation and formal time-dependent exposure assessment are warranted to confirm and extend these findings.

## Data Availability

The raw data supporting the conclusions of this article will be made available by the authors, without undue reservation.
