# Supplementary material for: Pathogen characteristics and pre-index clinical factors associated with multidrug-resistant organism infections in intensive care unit patients
Source: Front Public Health. 2026 Jul 30;14:1821168. doi: 10.3389/fpubh.2026.1821168 (PMC13469443; doi:10.3389/fpubh.2026.1821168)
Supplement: Supplementary file 1 [file Supplementary_file_1.DOCX]

Supplementary Table S1. Covariate balance before and after propensity score matching

| Covariate | Before matching MDRO (n=103) | Before matching non-MDRO (n=883) | SMD before matching | After matching MDRO (n=103) | After matching non-MDRO (n=206) | SMD after matching |
| --- | --- | --- | --- | --- | --- | --- |
| Age, years | 62.5 ± 10.9 | 65.8 ± 12.4 | 0.283 | 62.5 ± 10.9 | 63.6 ± 11.9 | 0.096 |
| Male sex, n (%) | 63 (61.2) | 481 (54.5) | 0.136 | 63 (61.2) | 119 (57.8) | 0.069 |
| Body mass index, kg/m² | 24.4 ± 2.8 | 23.7 ± 3.3 | 0.229 | 24.4 ± 2.8 | 24.3 ± 3.2 | 0.033 |
| Charlson Comorbidity Index | 3.2 ± 1.5 | 2.6 ± 1.6 | 0.387 | 3.1 ± 1.5 | 3.0 ± 1.6 | 0.064 |
| APACHE II score | 19.9 ± 6.5 | 16.8 ± 7.1 | 0.455 | 19.9 ± 6.5 | 18.0 ± 7.0 | 0.090 |
| SOFA score | 7.8 ± 3.2 | 6.0 ± 3.1 | 0.571 | 7.8 ± 3.2 | 6.9 ± 2.8 | 0.093 |
| Primary reason: infection, n (%) | 46 (44.7) | 320 (36.2) | 0.172 | 46 (44.7) | 93 (45.1) | 0.098 |
| Primary reason: postoperative care, n (%) | 30 (29.1) | 356 (40.3) | 0.237 | 30 (29.1) | 63 (30.6) | 0.032 |
| Primary reason: trauma, n (%) | 14 (13.6) | 84 (9.5) | 0.128 | 14 (13.6) | 24 (11.7) | 0.058 |
| Primary reason: neurocritical illness, n (%) | 13 (12.6) | 123 (13.9) | 0.039 | 13 (12.6) | 26 (12.6) | 0.000 |
| Shock, n (%) | 34 (33.0) | 188 (21.3) | 0.266 | 34 (33.0) | 64 (31.1) | 0.042 |
| Acute kidney injury, n (%) | 38 (36.9) | 200 (22.7) | 0.315 | 38 (36.9) | 60 (29.1) | 0.091 |
| Acute respiratory distress syndrome, n (%) | 17 (16.5) | 74 (8.4) | 0.248 | 17 (16.5) | 26 (12.6) | 0.095 |

Abbreviations: SMD, standardized mean difference; MDRO, multidrug-resistant organism; APACHE II, Acute Physiology and Chronic Health Evaluation II; SOFA, Sequential Organ Failure Assessment.

Supplementary Table S2. Missingness by MDRO infection status in the 1:2 matched cohort (n=309)

| Variable | MDRO infection (n=103), missing n (%) | Non-MDRO infection (n=206), missing n (%) | Absolute difference (percentage points) |
| --- | --- | --- | --- |
| Age | 0 (0.0) | 0 (0.0) | 0.0 |
| Sex | 0 (0.0) | 0 (0.0) | 0.0 |
| BMI | 3 (2.9) | 5 (2.4) | 0.5 |
| Charlson Comorbidity Index (CCI) | 1 (1.0) | 2 (1.0) | 0.0 |
| APACHE II (within 24 h) | 5 (4.9) | 9 (4.4) | 0.5 |
| SOFA (within 24 h) | 4 (3.9) | 9 (4.4) | 0.5 |
| WBC (within 24 h) | 2 (1.9) | 4 (1.9) | 0.0 |
| Procalcitonin (within 24 h) | 8 (7.8) | 16 (7.8) | 0.0 |
| C-reactive protein (within 24 h) | 6 (5.8) | 12 (5.8) | 0.0 |
| Lactate (within 24 h) | 3 (2.9) | 6 (2.9) | 0.0 |
| Albumin (within 24 h) | 6 (5.8) | 13 (6.3) | 0.5 |
| Pre-ICU antibiotic exposure | 0 (0.0) | 0 (0.0) | 0.0 |
| Pre-index ICU antibiotic exposure variables | 0 (0.0) | 0 (0.0) | 0.0 |
| Pre-index mechanical ventilation | 0 (0.0) | 0 (0.0) | 0.0 |
| Pre-index CRRT | 0 (0.0) | 0 (0.0) | 0.0 |

Abbreviations: BMI, body mass index; CCI, Charlson Comorbidity Index; APACHE II, Acute Physiology and Chronic Health Evaluation II; SOFA, Sequential Organ Failure Assessment; WBC, white blood cell count; CRRT, continuous renal replacement therapy.

Supplementary Table S3. Missingness summary and sensitivity analyses (matched cohort, n=309)

| Item | Result |
| --- | --- |
| Missingness range across covariates | 0.0%–7.8% |
| Variables with 0% missing | Age, sex, MDRO outcome, key microbiological fields |
| BMI missing | 8/309 (2.6%) |
| APACHE II missing | 14/309 (4.5%) |
| SOFA missing | 13/309 (4.2%) |
| WBC missing | 6/309 (1.9%) |
| Procalcitonin missing | 24/309 (7.8%) |
| CRP missing | 18/309 (5.8%) |
| Lactate missing | 9/309 (2.9%) |
| Albumin missing | 19/309 (6.1%) |
| Complete-case sample size | 285/309 (92.2%) |
| MDRO/non-MDRO in complete-case set | 95 / 190 |
| Excluded due to missing model covariates | 24 (7.8%) |
| Concordance of key model variables (CCA vs MI) | Direction consistent for all; statistical significance unchanged for all key model variables |
| Conclusion after excluding missing cases | Main conclusions unchanged |

Abbreviations: CCA, complete-case analysis; MI, multiple imputation; WBC, white blood cell count; CRP, C-reactive protein.
